# Supplementary figures and images for: Efficacy and safety of metabolic interventions for the treatment of severe COVID-19: in vitro, observational, and non-randomized open-label interventional study
Source: eLife. 2023 Jan 27;12:e79946. doi: 10.7554/eLife.79946 (PMC9937660; doi:10.7554/eLife.79946)

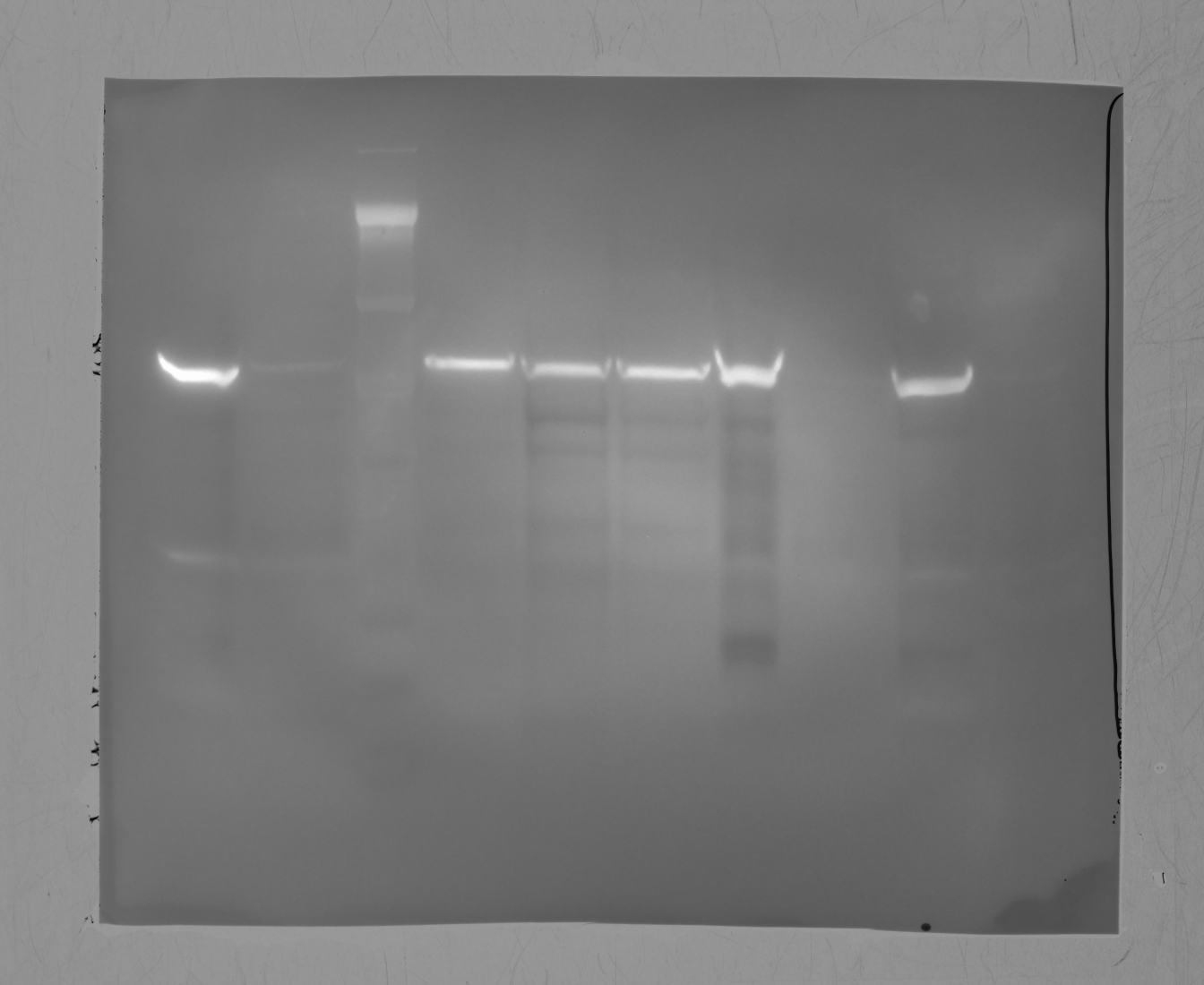

Supplement: Figure 3—figure supplement 3—source data 1. — (1) original and (2) inverted files of the full raw unedited alpha tubulin blot. (3) original and (4) inverted files of the full raw unedited PPAR alpha blot. (5) The uncropped blots with the relevant bands are clearly labeled. [file elife-79946-fig3-figsupp3-data1.zip › Raw_Blots/1 -5sec w lad.Tif]

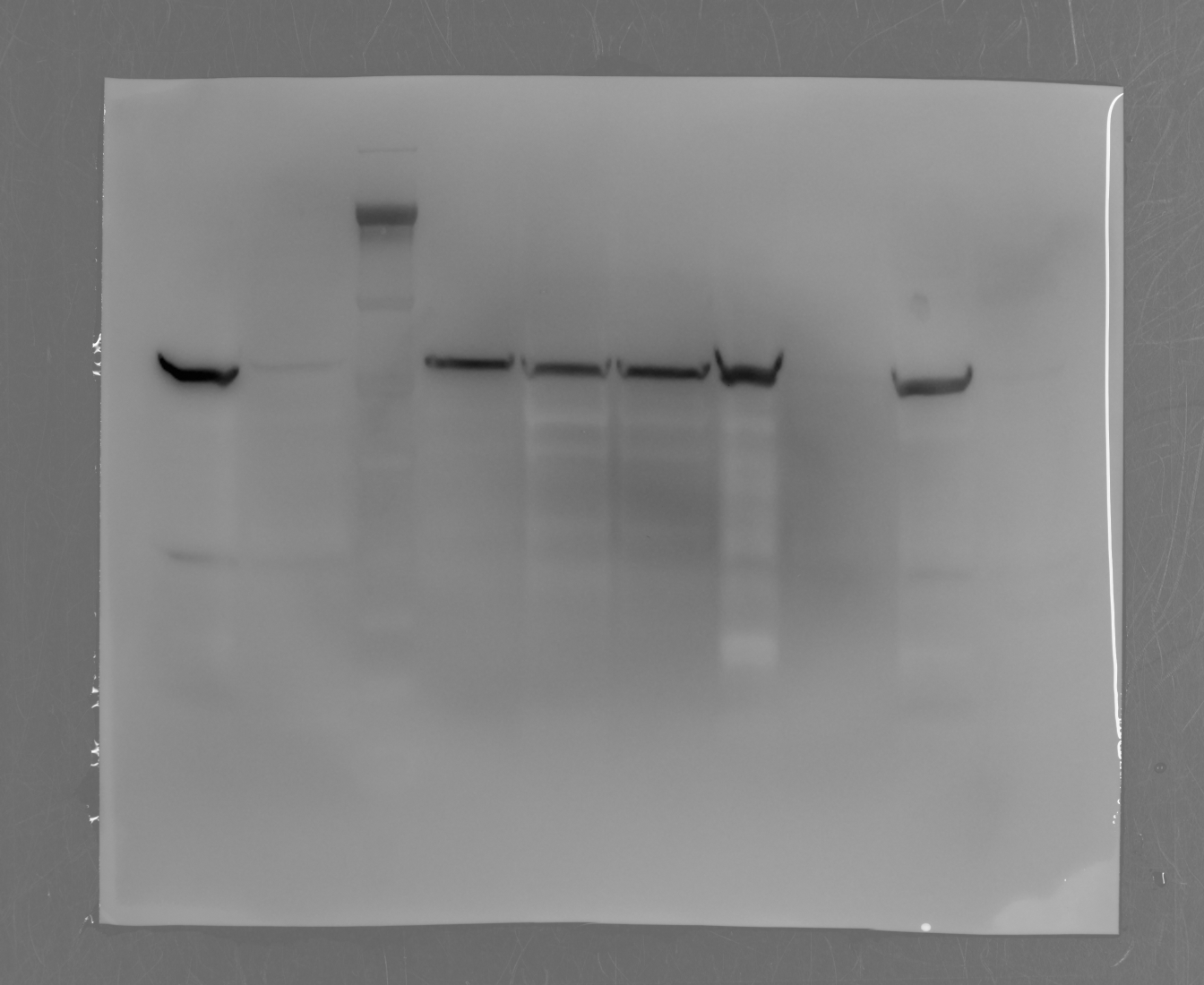

Supplement: Figure 3—figure supplement 3—source data 1. — (1) original and (2) inverted files of the full raw unedited alpha tubulin blot. (3) original and (4) inverted files of the full raw unedited PPAR alpha blot. (5) The uncropped blots with the relevant bands are clearly labeled. [file elife-79946-fig3-figsupp3-data1.zip › Raw_Blots/2 -5sec w lad__inverted.Tif]

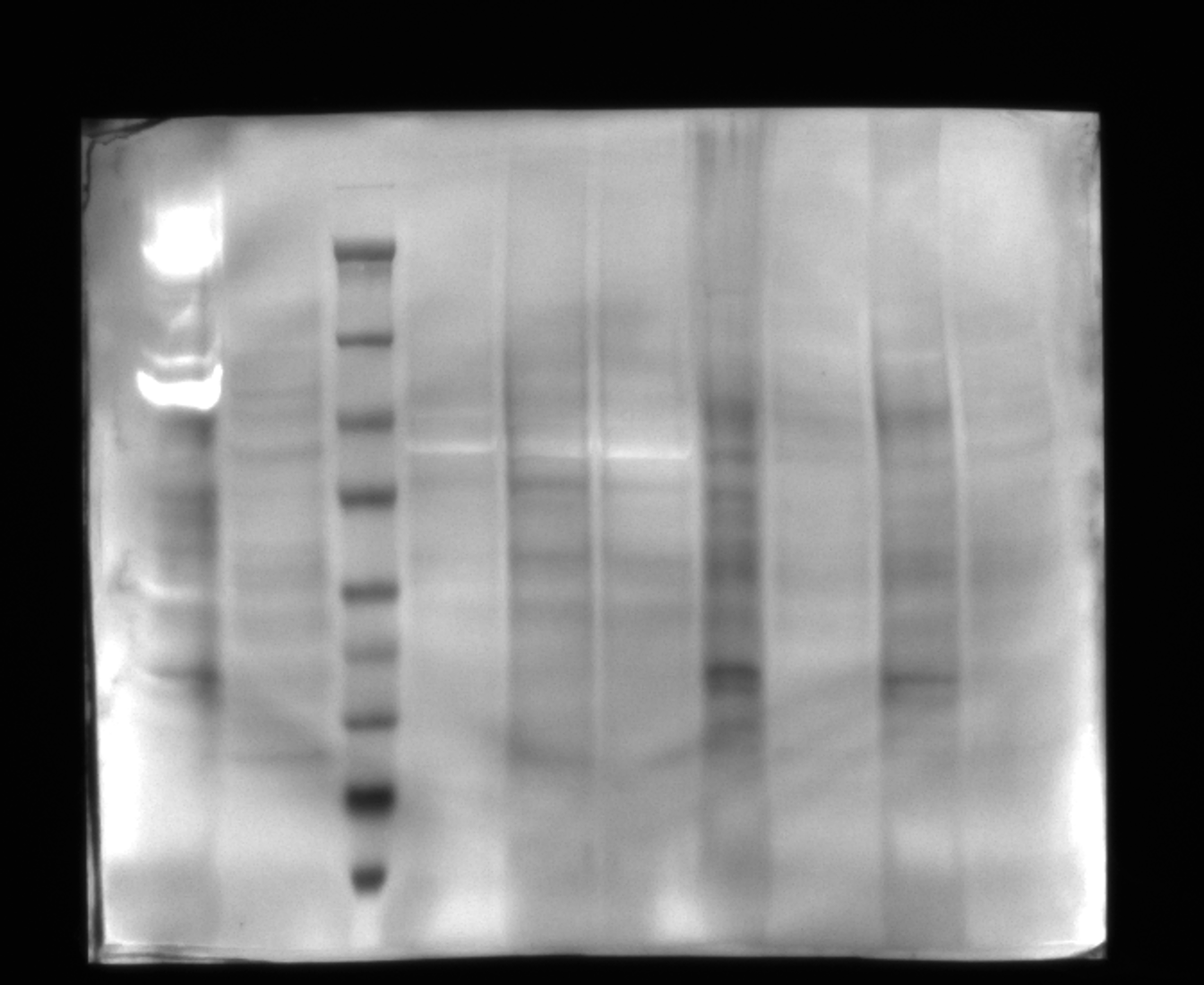

Supplement: Figure 3—figure supplement 3—source data 1. — (1) original and (2) inverted files of the full raw unedited alpha tubulin blot. (3) original and (4) inverted files of the full raw unedited PPAR alpha blot. (5) The uncropped blots with the relevant bands are clearly labeled. [file elife-79946-fig3-figsupp3-data1.zip › Raw_Blots/3 -22sec.Tif]

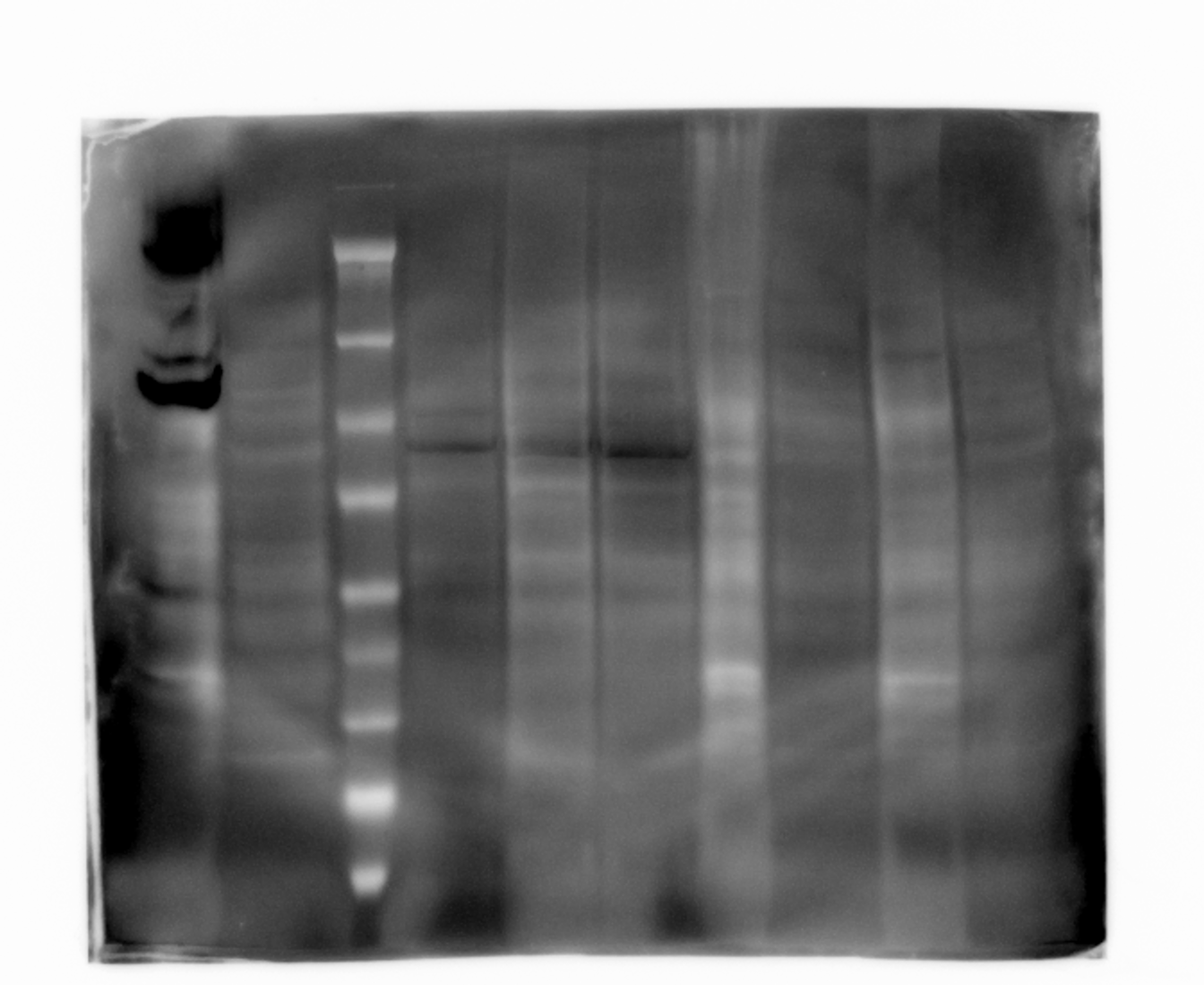

Supplement: Figure 3—figure supplement 3—source data 1. — (1) original and (2) inverted files of the full raw unedited alpha tubulin blot. (3) original and (4) inverted files of the full raw unedited PPAR alpha blot. (5) The uncropped blots with the relevant bands are clearly labeled. [file elife-79946-fig3-figsupp3-data1.zip › Raw_Blots/4 -22sec__inverted.tif]

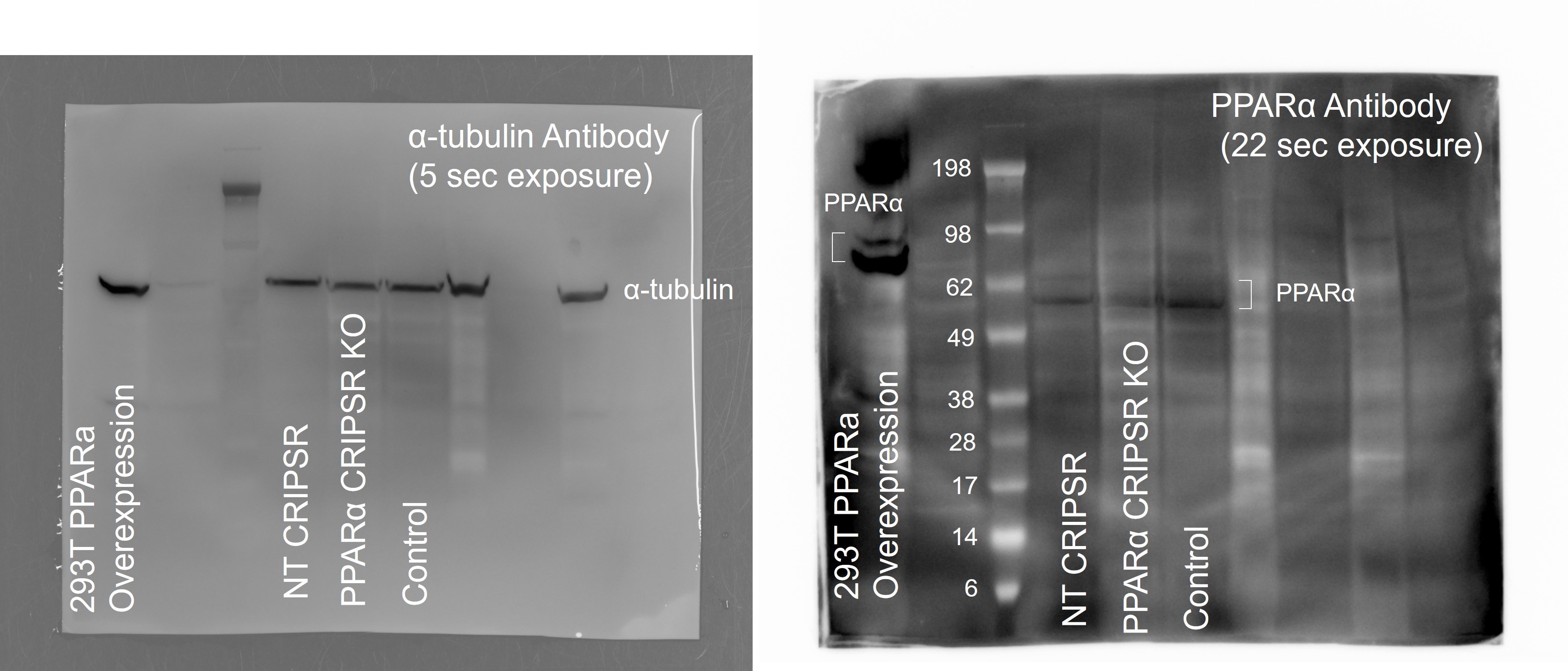

Supplement: Figure 3—figure supplement 3—source data 1. — (1) original and (2) inverted files of the full raw unedited alpha tubulin blot. (3) original and (4) inverted files of the full raw unedited PPAR alpha blot. (5) The uncropped blots with the relevant bands are clearly labeled. [file elife-79946-fig3-figsupp3-data1.zip › Raw_Blots/5 - uncropped gels or blots with the relevant bands clearly labelled.jpg]
